# Vascular‐specific expression of *Gastrodia* antifungal protein gene significantly enhanced cotton *Verticillium* wilt resistance

**DOI:** 10.1111/pbi.13308

**Published:** 2020-01-04

**Authors:** Yiqin Wang, Chengzhen Liang, Shenjie Wu, Guiliang Jian, Xueyan Zhang, Huanyang Zhang, Jiuyou Tang, Jing Li, Gaili Jiao, Fuguang Li, Chengcai Chu

**Affiliations:** ^1^ State Key Laboratory of Plant Genomics Institute of Genetics and Developmental Biology, the Innovative Academy of Seed Design Chinese Academy of Sciences Beijing China; ^2^ Biotechnology Research Institute Chinese Academy of Agricultural Sciences Beijing China; ^3^ Research Center of Biotechnology Shanxi Academy of Agricultural Sciences Taiyuan Shanxi province China; ^4^ Institute of Plant Protection Chinese Academy of Agricultural Sciences Beijing China; ^5^ Institute of Cotton Research Chinese Academy of Agricultural Sciences Anyang Henan province China; ^6^ Cotton Research Institute Shanxi Academy of Agricultural Sciences Yuncheng Shanxi province China

**Keywords:** vascular‐specific promoter, glycine decarboxylase‐P promoter, *Gastrodia* antifungal protein, cotton *Verticillium* wilt resistance, methyl jasmonate and salicylic acid responsive elements, wounding induction

## Introduction

As the most important natural fibre crop in the world, upland cotton (*Gossypium hirsutum* L.) accounts for >95% of the world’s cotton production. However, most of the current upland cotton cultivars are susceptible to *Verticillium* wilt, a major fungal disease termed as the ‘cancer of cotton’, which exists in cotton‐producing areas worldwide and causes huge economic losses every year (Li *et al.*, 2019). Currently, selecting and breeding cultivars with broad‐spectrum resistance to *Verticillium* wilt are considered to be one of the most effective approaches for controlling this disease. In the past two decades, there were many studies on the genetic engineering of cotton *Verticillium* wilt resistance using resistance genes cloned from cotton or other plant species, and even functional genes from the pathogen like *V. dahliae* and *Xanthomonas oryzae pv. oryzae* (Wang *et al.*, 2004; Zhang *et al.*, 2018). However, most of these studies have focused on gene function analysis using transgenic *Arabidopsis*, tomato or tobacco, and only a few genes have been shown to increase the resistance of transgenic cotton to *Verticillium* wilt (Jun *et al.*, 2015; Li *et al.*, 2019).

For more than 20 years, our group has studied the *Gastrodia* antifungal protein (GAFP) family and their functions in *Verticillium* wilt resistance of transgenic cotton (Wang *et al.*, 2016). We found that expression of *GAFPs* in cotton could significantly enhance *Verticillium* wilt resistance and *GAFP4* is an excellent target gene for genetic engineering to control *Verticillium* wilt. Previously, we utilized the cauliflower mosaic virus (CaMV) *35S* constitutive promoter to drive the *GAFP* genes, while the persistent and stable expression of exogenous genes in all plant tissues raises concerns about potential cost of possible metabolic disorders and the excessive consumption of intracellular substances and energy, and further the expression of multiple exogenous genes under the control of the same promoter may also cause gene silencing or co‐inhibition (Zheng *et al.*, 2007). Therefore, it will be desirable to use tissue‐specific or stage‐specific promoter for particular application of genetic engineering. Generally, the fungal pathogen *Verticillium dahliae* infects the plant *via* the roots and then spreads through the vascular tissue. The infection finally blocks water uptake and causes leaf yellowing, necrosis, defoliation and more severely the death of whole plant (Zhou *et al.*, 2017). Therefore, it will be desirable to express the *GAFP4* gene under the control of a vascular‐specific promoter, which may enhance the level of resistance to *Verticillium* wilt without penalty on plant growth and development.

**Figure 1:**
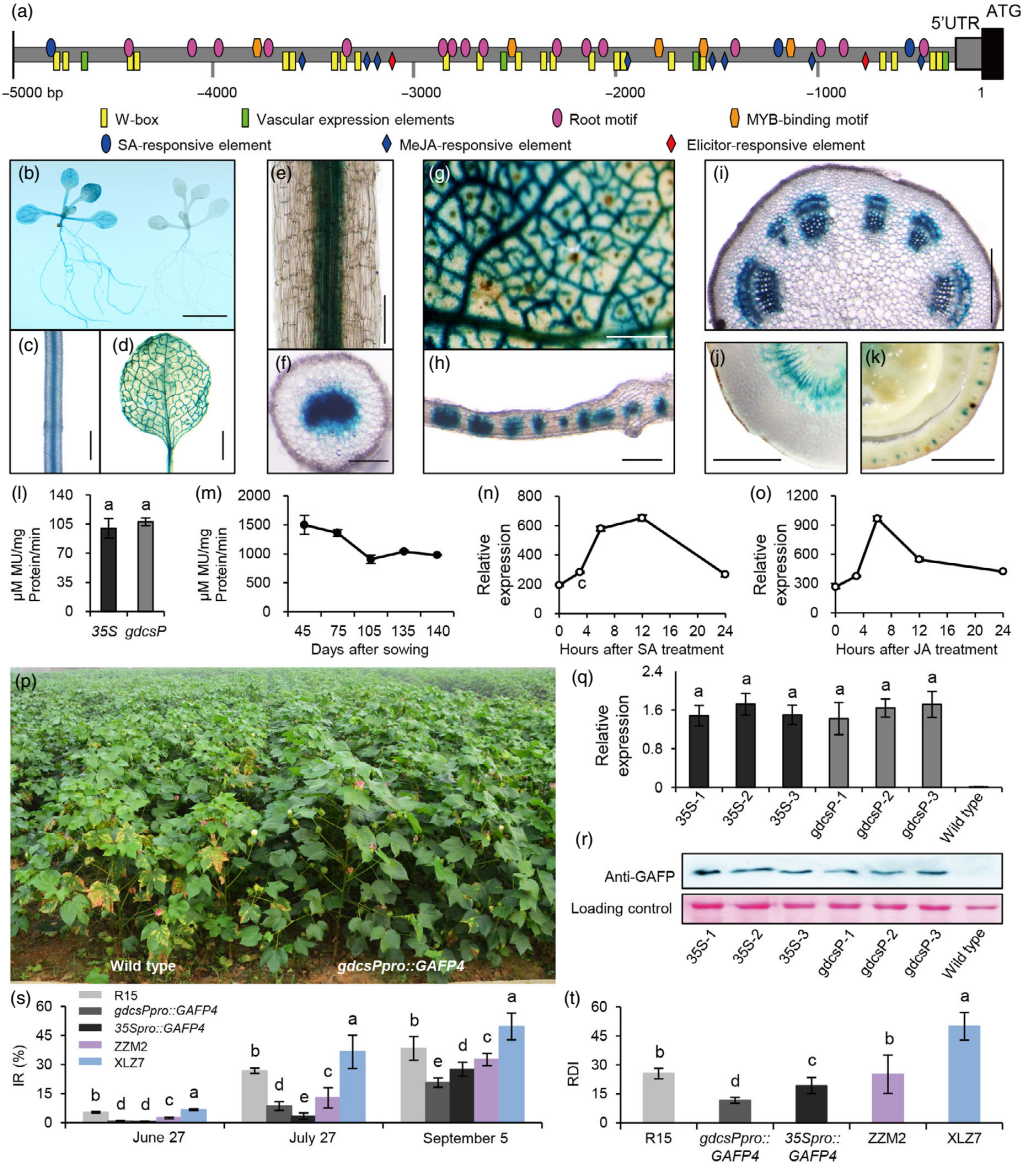
Vascular‐specific expression of the *gdcsP* promoter and its application in *GAFP4* transgenic cotton. (a) A map of *cis*‐acting regulatory elements in *gdcsP* promoter from analysis using bioinformatics tools Sogo (https://sogo.dna.affrc.go.jp) and PlantCARE (http://bioinformatics. psb.ugent.be/webtools/plantcare). (b‐d) Expression of the *gdcsP* promoter in *Arabidopsis* seedlings. (b): whole plant of transgenic (left) and control (right), c: root; d: leaf. (e‐k) Expression of the *gdcsP* promoter in transgenic cotton. e‐f: root; g: young leaf; h: mature leaf; i: young stem; j: petiole; k: young boll. (l) Comparison of the activity of CaMV *35S* promoter with the *gdcsP* promoter in transgenic *Arabidopsis*. For each promoter, four transgenic lines with similar *GUS* expression level and six individual plants per line are used. (m) Activity of the *gdcsP* promoter at different growth stages of transgenic cotton. Three transgenic lines with similar *GUS* expression level and six individual plants per line are used. (n‐o) Induction of the *gdcsP* promoter by phytohormones. n: 50 μm SA; o: 50 μM Me‐JA. Each treatment group included 5 individual seedlings. (p) The evaluation of *gdcsP_Pro_::GAFP4* transgenic cotton in the *Verticillium* wilt nursery in Anyang. (q‐r) The molecular evidence of transgenic cotton. Three lines with similar expression of *GAFP4* (q) and protein content (r) were selected from different promoter transgenic plants for further field test in Langfang. (s‐t) The evaluation of *GAFP4* transgenic cotton in the *Verticillium* wilt nursery in Langfang. The resistant and susceptible control is ZhongZhiMian‐2 (ZZM‐2) and XinLuZao‐7 (XLZ‐7), respectively. Each transgenic line has 3 random repetitions, and each repetition has more than 30 individual plants. The disease surveys were conducted at seedling stage, flowering stage and bolling stage, respectively, when plants were growing for about 8, 12 and 16 weeks after germination. The disease index (DI) was calculated according to the following formulas: DI = [(Σdisease score × amount of infected plants)/total checked plants × 4] × 100. Relative DI (RDI) = DI × K (correction factor, K = 50.0/DI‐sc, sc: susceptible control). Different letters indicate significant difference according to Duncan’s multiple range tests (*P *< 0.05).

Previously, we found that the promoter of *Glycine decarboxylase* (*GDC*) *P‐protein* subunit (*gdcsP*) from the C_3_–C_4_ intermediate plant *Flaveria anomala* has vascular bundle‐sheath‐specific expression pattern (Chu, 1996). Furthermore, it also has been shown to have vascular‐specific expression in monocotyledonous rice (Chen *et al.*, 2001). Using bioinformatics tools, we analysed the nearly 5 kb regions of *gdcsP* promoter. We found 4 elements required for vascular expression and 16 root motifs that required for strong root‐specific expression. Moreover, the *gdcsP* promoter also contains 8 methyl jasmonate (Me‐JA) and 3 salicylic acid (SA)‐responsive elements, and few elicitor‐induced, defence‐related WRKY binding site (W‐box) and the MYB binding motif (Figure 1a). Therefore, the *gdcsP* promoter could respond to both pathogen infection and wounding (Nishiuchi *et al.*, 2004), which would more effectively inhibit the fungal pathogen at a very early stage with the *gdcsP* promoter, as the pathogen *V. dahliae* normally infects cotton roots through wounding. Therefore, the *gdcsP* promoter should be an ideal promoter for genetic engineering of cotton *Verticillium* wilt resistance. To examine whether the *gdcsP* promoter is active in different dicotyledonous plants, we used it to drive the expression of *β‐glucuronidase* (*GUS*) reporter gene (*gdcsP_Pro_::GUS*) in *Arabidopsis* and upland cotton variety R15. In *Arabidopsis*, the *gdcsP* promoter could drive vascular‐specific *GUS* expression in different parts of the plant at a level similar to that of the CaMV *35S* promoter (Figure 1b‐d and l). In transgenic cotton seedlings, strong GUS activity was observed in the vascular bundles of the root, stem, leaf, petiole and even young bolls (Figure 1e‐k). More importantly, the activity of *gdcsP* promoter could keep at high level throughout the entire cotton life cycle (Figure 1m). Furthermore, the *gdcsP* promoter could also be induced by SA and Me‐JA (Figure 1n‐o), suggesting that it would be a very potential promoter used for manipulation of biotic stress resistance.

We further constructed the *gdcsP_Pro_::GAFP4* vector and transformed it into upland cotton variety R15. In Anyang (35°12′N, 113°37′E), Henan province, the transgenic plants had significantly higher level of resistance compared with the non‐transgenic controls in the *Verticillium* wilt disease nursery (Figure 1p). To compare the efficiency of *gdcsP* promoter with CaMV *35S* promoter, three homozygous *gdcsP_Pro_::GAFP4* transgenic lines, together with three *35S_Pro_::GAFP4* transgenic lines with similar expression level of *GAFP4* and the content of antifungal protein (Figure 1q‐r), were selected for further field evaluation in *Verticillium* wilt disease nursery in Langfang (39°56′N, 116°20′E), Hebei Province. From the result of surveys, we observed that all *GAFP4* transgenic lines performed better than the non‐transgenic control lines in this large scale evaluation. The *gdcsP_Pro_::GAFP4* transgenic lines have lower infection rate (IR) and relative disease index (RDI) than the *35S_Pro_::GAFP4* transgenic lines in the last survey, which is even better than the resistance control ZhongZhiMian‐2 (ZZM‐2) (Figure 1s‐t). These results demonstrate that vascular‐specific expression of the *GAFP4* is more effective than constitutive expression in conferring *Verticillium* wilt disease resistance. From the result of GUS staining, the strong and specific expression of *gdcsP_pro_* in vascular tissue was observed (Figure 1e‐k). Therefore, with the similar expression level of *GAFP*, the antifungal protein in the *gdcsP_pro_::GAFP4* transgenic plants would be more concentrated in the vascular tissue, leading to more effective disease resistance compared with the transgenic plants with constitutive expression of *GAFP4*. Therefore, the strong vascular‐specific activity of the *gdcsP* promoter makes it an effective tool for genetic engineering of *Verticillium* wilt resistance in cotton.

Since the *gdcsP* promoter is active in both monocot and dicot plants, this promoter also has great potential in the genetic engineering of other disease species. For example, as it can express in young bolls (Figure 1k), the *gdcsP* promoter could be used for engineering resistance to cotton boll diseases such as bollworm and phytophthora boll rot, etc. Similar to the fungal pathogen *V. dahliae*, plant viruses need to infect the vascular tissue to spread in the entire plant, and most piercing‐sucking insects also sucked the nutrients by inserting their mouthparts into the vascular tissue. Therefore, the vascular‐specific promoters such as the *gdcsP* promoter could have great potential and broad application in genetic engineering of virus‐ and insect‐resistant plants.

## Conflict of interests

The authors have no conflict of interests.

## Author contributions

Y.W. and C.C. designed experiments; S.W. H.Z., and J.L. performed cotton transformations; S.W., Ga.J., Gu.J., X.Z., F.L., C.L., J.T. and Y.W. performed field trials; Y.W., C.C. and C.L. performed data analysis and wrote the paper.
